# Supplementary material for: Learning to detect AI texts and learning the limits
Source: PLoS One. 2025 Oct 15;20(10):e0333007. doi: 10.1371/journal.pone.0333007 (PMC12527182; doi:10.1371/journal.pone.0333007)
Supplement: S2 File — All data and scripts used in the study, some additional descriptive statistics, scalable formate figures. (ZIP) [file pone.0333007.s002.tgz › data and scripts/OSF/scripts/analysis/Demographics/distribution_Demographics-ai_usage.pdf]

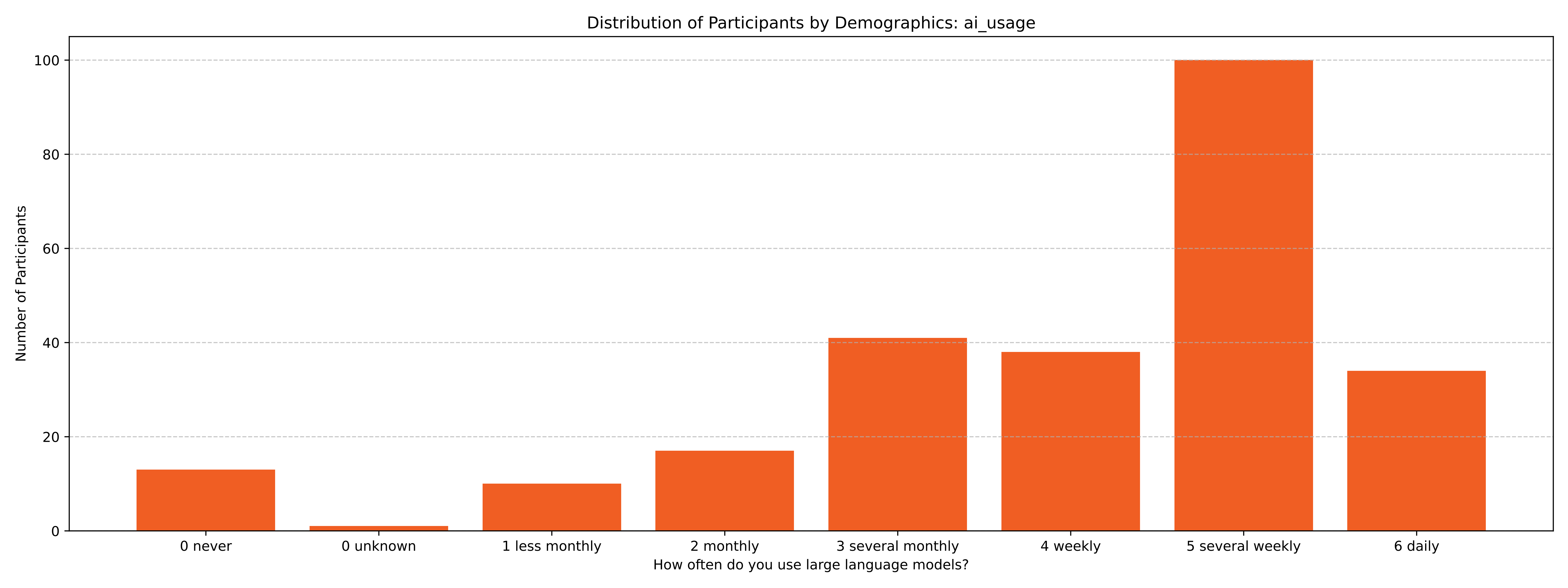
Distribution of Participants by Demographics: ai_usage
Number of Participants
0
20
40
60
80
100
0 never
0 unknown
1 less monthly
2 monthly
3 several monthly
4 weekly
5 several weekly
6 daily
How often do you use large language models?
